# Lung glutathione adaptive responses to cigarette smoke exposure

**DOI:** 10.1186/1465-9921-12-133

**Published:** 2011-10-07

**Authors:** Neal S Gould, Elysia Min, Steve Gauthier, Richard J Martin, Brian J Day

**Affiliations:** 1 Department of Medicine, University of Colorado, Denver, CO, USA; 2 Department of Immunology, University of Colorado, Denver, CO, USA; 3 Department of Pharmaceutical Sciences, University of Colorado, Denver, CO, USA; 4 Department of Medicine, National Jewish Health, Denver, CO, USA

## Abstract

**Background:**

Smoking tobacco is a leading cause of chronic obstructive pulmonary disease (COPD), but although the majority of COPD cases can be directly related to smoking, only a quarter of smokers actually develop the disease. A potential reason for the disparity between smoking and COPD may involve an individual's ability to mount a protective adaptive response to cigarette smoke (CS). Glutathione (GSH) is highly concentrated in the lung epithelial lining fluid (ELF) and protects against many inhaled oxidants. The changes in GSH that occur with CS are not well investigated; therefore the GSH adaptive response that occurs with a commonly utilized CS exposure was examined in mice.

**Methods:**

Mice were exposed to CS for 5 h after which they were rested in filtered air for up to 16 h. GSH levels were measured in the ELF, bronchoalveolar lavage cells, plasma, and tissues. GSH synthesis was assessed by measuring γ-glutamylcysteine ligase (GCL) activity in lung and liver tissue.

**Results:**

GSH levels in the ELF, plasma, and liver were decreased by as much as 50% during the 5 h CS exposure period whereas the lung GSH levels were unchanged. Next, the time course of rebound in GSH levels after the CS exposure was examined. CS exposure initially decreased ELF GSH levels by 50% but within 2 h GSH levels rebound to about 3 times basal levels and peaked at 16 h with a 6-fold increase and over repeat exposures were maintained at a 3-fold elevation for up to 2 months. Similar changes were observed in tissue GCL activity which is the rate limiting step in GSH synthesis. Furthermore, elevation in ELF GSH levels was not arbitrary since the CS induced GSH adaptive response after a 3d exposure period prevented GSH levels from dropping below basal levels.

**Conclusions:**

CS exposures evoke a powerful GSH adaptive response in the lung and systemically. These data suggests there may be a sensor that sets the ELF GSH adaptive response to prevent GSH levels from dipping below basal levels. Factors that disrupt GSH adaptive responses may contribute to the pathophysiology of COPD.

## Introduction

The lung is unique since it is exposed to high ambient oxygen levels and a constantly changing atmospheric environment. The lung is exposed on a daily basis to a wide range of oxidants ranging from ozone, smog, diesel exhaust, dust particles and cigarette smoke (CS). In comparison to all other forms of inhaled oxidants, CS may be one of the most prevalent and preventable oxidant exposures, with nearly 1 billion smokers worldwide. CS contains at least 4,800 different chemicals with over 10^14 ^radicals per puff that can cause DNA, protein, and lipid oxidation among many other effects [1-3]. Fortunately, the lung has developed adaptive mechanisms to defend itself against inhaled oxidants [4].

CS is the primary cause of chronic obstructive pulmonary disease (COPD), with as much as 90% of COPD patients having been smokers at one point in their lifetime [5,6]. However, a conundrum exists in that not all smokers go on to develop COPD. It has been estimated that only about 25% of smokers develop COPD, typically later in life [7]. The fact that 75% of smokers do not develop COPD points to strong defense mechanisms to handle the increased oxidant burden that smoking puts on the lung. Furthermore, the effects of CS are not limited only to the lung. CS has been implicated in several cardiovascular diseases, liver diseases as well cancer [8,9].

One of the primary lung defenses against CS is the epithelial lining fluid (ELF) [4]. The ELF is a thin continuous fluid that hydrates the epithelial cells throughout the airways. The ELF is comprised of a heterogeneous mixture of mucus, cells, proteins, and low molecular weight antioxidants [4]. At its most basic, the ELF provides a physical barrier against many inhaled oxidants and an important component of host defense against pathogens. In addition to being able to act as a physical barrier, there are high concentrations of antioxidants within the ELF that act to detoxify exogenous or endogenous oxidants [4,10,11]. One of these antioxidants is glutathione (GSH) which is concentrated in the ELF 10-100 times more than in the plasma [12]. GSH is a tripeptide comprised of glutamate, cysteine, and glycine and is synthesized and utilized in every organ throughout the body. γ-Glutamylcysteine ligase (GCL) is the rate limiting enzyme involved in GSH synthesis and its expression has been shown to be induced in response to CS [13]. GSH can react with a wide range of molecules, making it an effective antioxidant at detoxifying many of the diverse reactive electrophilic components of CS.

CS has been shown to induce a number of different antioxidant defenses including the expression of enzymes and transcription factors that lead to increased synthesis of GSH [14]. This increase GSH response to CS is referred to as the GSH adaptive response. Despite being a potentially critical antioxidant, not much is known about the acute effects of CS on the GSH adaptive response and how GSH levels can fluctuate both during and between CS exposures especially in the lung ELF. Therefore the present study sought to characterize the changes in GSH with exposure to CS in various compartments with an emphasis on the changes that occur in the ELF both during and between acute CS exposures in vivo.

## Methods

### Cigarette smoke extract (CSE) preparation

The smoke of one 3R4F Kentucky reference cigarette was bubbled through 7 mL of room temperature PBS. The extract was measured spectrophotometrically at a wavelength of 210 nm, an absorbance of 1.3-2.0 was considered acceptable. The resulting extract was deemed 100% CSE and was diluted in normal cell media.

### In vitro CSE exposure

Human bronchial epithelial cell line (16HBE, ATCC) was grown in DMEM supplemented with glutamine, 10% FBS and antibiotics. Cells were grown to roughly 85% confluence and CSE was diluted in the media to a final concentration of 20%. The cells were exposed for various times, after which the media was removed and the cells were washed with warm PBS and lysed by brief sonication in fresh PBS. Media and lysate was stored at -20°C until analysis. Cytotoxicity was assessed by measuring lactate dehydrogenase release as previously described [15] and CSE treated cells under conditions tested did not have changes in cell viability that were statistically significantly different from controls and was greater than 85%.

### In vivo cigarette smoke (CS) exposure

Male C57B/6 two month old mice were obtained from Jackson's laboratory (Bar Harbor, ME). The mice were exposed to CS from Kentucky reference cigarette 3R4F (University of Kentucky) for 5 h/day. The average particulate matter was 100 mg/m^3 ^and carbon monoxide levels were less than 350 ppm. Unless explicitly stated the mice were sacrificed 16 h after the CS exposure via cardiac exanguastion and bronchoalveolar lavage (BAL) was performed using two 750 μL rinses of cold isotonic potassium phosphate solution. The dilution of the ELF was calculated by measuring urea in both the BAL fluid (BALF) and plasma as previously reported [16]. All animal procedures followed the Public Health Service Policy on Humane Care and Use of Laboratory Animals and received prior approval by the National Jewish Health IACUC committee.

### Tissue collection

Both BALF and whole blood were kept on ice and then centrifuged to separate the BAL cells and plasma, respectively. The lungs and liver were perfused using PBS and snap frozen in liquid nitrogen. Tissues were stored at -80°C until analysis. For tissue analysis, roughly 25 mg tissue was homogenized in 0.5 mL potassium phosphate buffer. The resulting solution was clarified of cell debris by centrifugation and analyzed for GSH levels or GCL activity.

### Measurement of glutathione

Total glutathione (GSH) was measured spectrophotometrically in the BALF, plasma, and tissues as previously described [17]. GSH was measured by adding the standard or sample to 100 μL of a 1:1 mixture of 3 units/mL glutathione reductase with 0.67 mg/mL 5,5'-Dithiobis(2-nitrobenzoic acid) (DTNB). The reaction was initiated by the addition of 20 μL of 0.67 mg/mL NADPH and the increase in absorbance at 412 nm was monitored. Values measured in BALF were normalized to urea, values in tissues were normalized to protein content. For in vitro samples both the media and lysate were normalized to the lysate protein. The limit of detection for GSH was 0.2 μM.

### Measurement of γ-glutamylcysteine ligase (GCL) activity

GCL activity was measured in lung and liver homogenates as previously described [18]. Samples were split for baseline and GCL activity measurements. Sample or standard was added to GCL reaction buffer (400 mM Tris, 40 mM ATP, 20 mM L-glutamic acid, 2.0 mM EDTA, 20 mM sodium borate, 2 mM serine, and 40 mM MgCl_2_). The reaction was initiated using 2 mM cysteine added to the GCL activity samples and allowed to incubate for 30 min. The reaction was halted by the addition of sulfosalicylic acid and protein precipitate was removed by centrifugation. Standard or sample was transferred in triplicate to a 96-well plate and GSH was then derivitized by the addition of 10 mM 2,3-naphthalenedicarboxyaldehyde and the fluorescence at Ex/Em wavelength of 472/528 nm was quantified using a fluorescent plate reader (Synergy 2, BioTek). GCL activity was calculated as the difference in GSH concentration between the reaction and baseline samples per minute normalized to the protein content in the sample.

### Statistics

Data represented as mean ± standard error with an n = 5 for each group. One way analysis of variance with Newman-Keuls post test for multiple comparisons was run using Prism 5 software (Graphpad). Different letters above each group denotes a significant difference (p < 0.05) between groups.

## Results

### Airway epithelial cells export GSH in response to CSE over time

**Figure 1:**
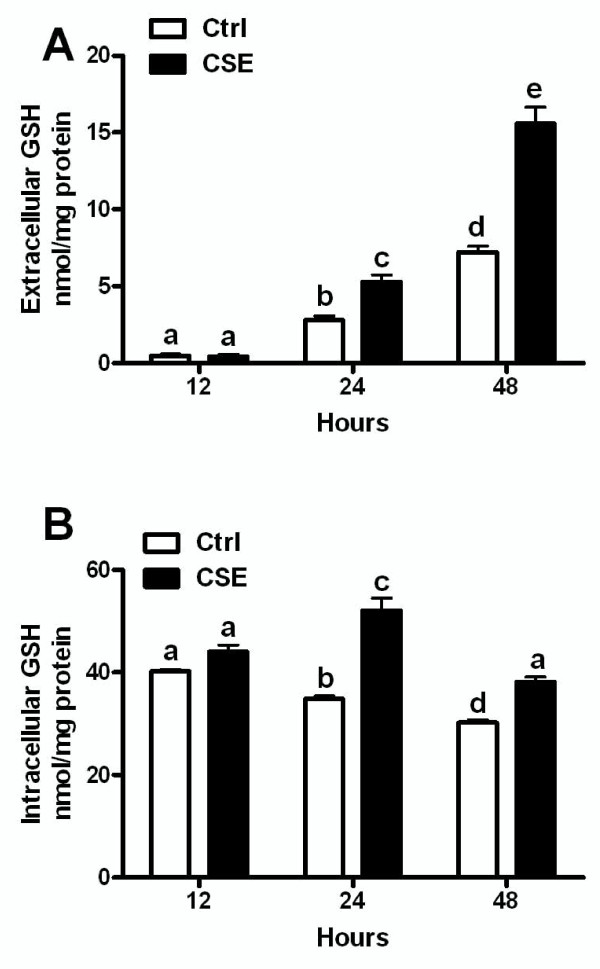
**Airway cells export GSH in response to CSE over time**. The extracellular (A) and intracellular (B) GSH levels after the exposure of 16HBE cells to 20% CSE at 12, 24, and 48 hours. Data represented as mean ± SEM, with different letters representing statistically significant differences between groups, p < 0.05.

Bronchial epithelial cells were exposed to CSE for 12, 24, and 48 hours to determine whether cells retain or export GSH in response to the CSE exposure. No change in extracellular GSH levels were observed after 12 hours of CSE exposure, but by 24 and 48 hours there was significantly more extracellular GSH with CSE exposure versus the control (Figure 1A). There was no change in viability between the control and CSE exposed cells at any timepoint (data not shown), which supports cells actively exporting GSH rather than it being released due to cell lysis. Additionally, the intracellular GSH was not changed at 12 hours between control and CSE but by 24 and 48 hours there was significantly increased intracellular GSH in the CSE exposed cells (Figure 1B).

### Lung ELF GSH is maintained over repeated CS exposures

**Figure 2:**
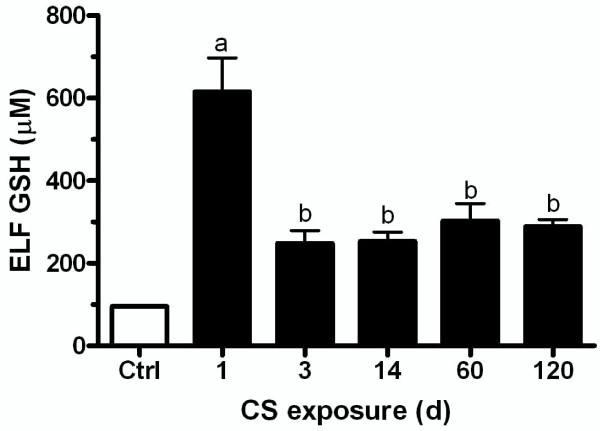
**Epithelial lining fluid (ELF) GSH levels with repeated CS exposures**. Mice were exposed to CS for 5 days/week between 1 and 120 days. ELF GSH levels are highest after 1 day of CS compared to repeated exposures. Data represented as mean ± SEM, with different letters representing statistically significant differences between groups, p < 0.05.

Mice were exposed to CS for 5 h/d for various times to determine when the ELF GSH adaptive response was maximal (Figure 2). The GSH adaptive response in the ELF peaked after one day of CS exposure, with nearly 600 μM GSH in the ELF. By day 3, the ELF GSH was actually less than the first day at only 250 μM. The same GSH levels after 3 days was maintained in the ELF over extended exposures up to 120 days. Due to the lack of major changes in ELF GSH with longer exposures, it stands to reason that the GSH adaptive response was established early, within 1 day, and then maintained for extended periods of time.

### CS exposure acutely depletes GSH levels

**Figure 3:**
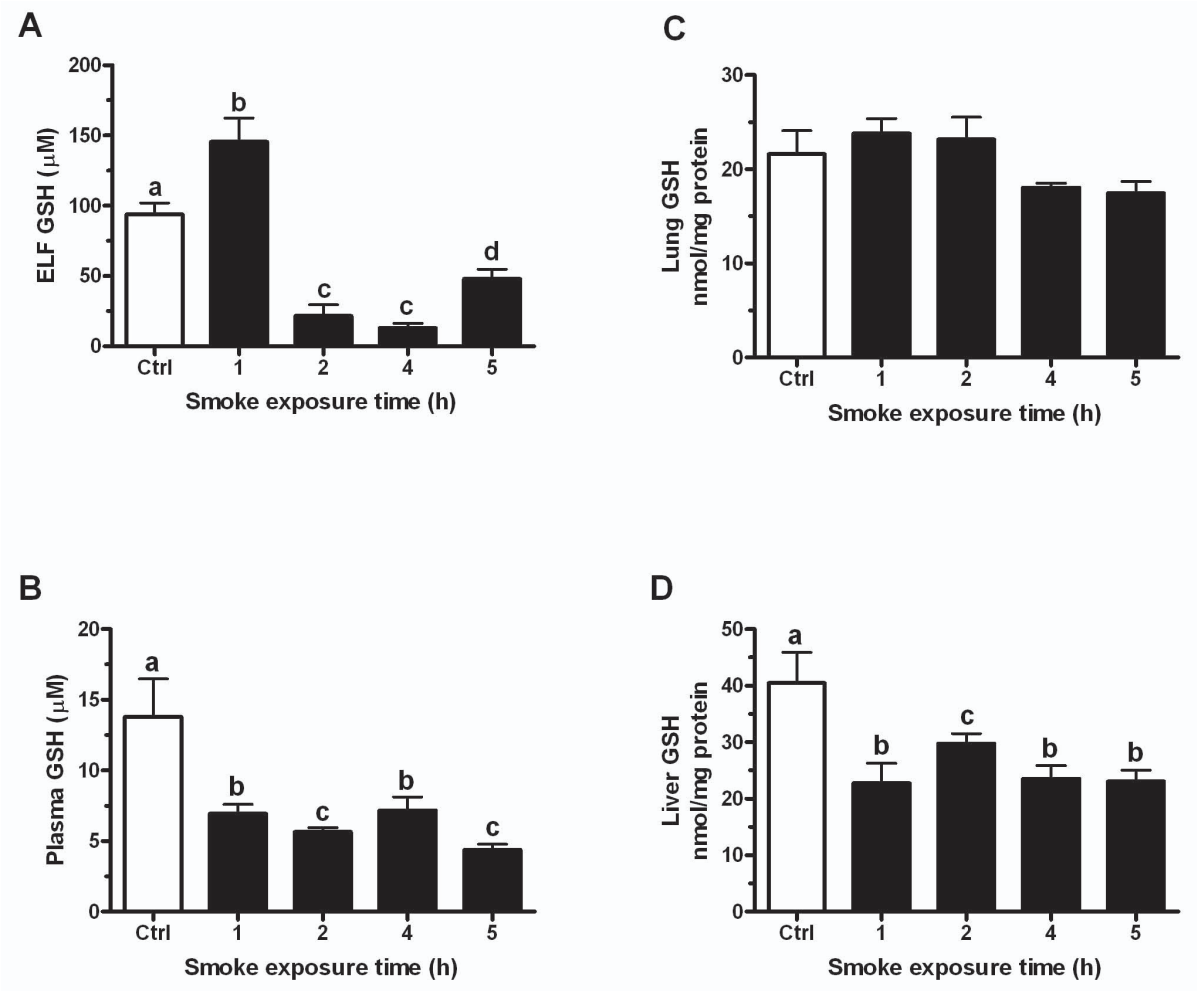
**Changes in GSH levels that occur during the CS exposure**. Mice were exposed to CS for 1, 4 or 5 hours and sacrificed immediately following exposure. ELF (A), plasma (C), and liver (D) GSH levels fell during the CS exposure while lung (B) GSH levels were maintained throughout the exposure. Data represented as mean ± SEM, with different letters representing statistically significant differences between groups, p < 0.05.

We sought to determine the effect of CS exposure on the GSH levels before the adaptive response had been established since many of the GSH changes are established within an acute timeframe. Mice were exposed to CS for 1, 4, or 5 hours and then sacrificed immediately following the smoke exposure. CS exposure depleted ELF GSH levels by 4 hours from 100 μM to 25 μM, which was roughly the level where it remained until the end of the CS exposure (Figure 3A). Furthermore, in both the plasma (Figure 3C) and liver (Figure 3D) there was a 50% decrease in GSH levels following all of the CS exposure times. Interestingly, the lung GSH (Figure 3B) was maintained at the same level throughout the CS exposure.

### GSH levels rebound between CS exposures

**Figure 4:**
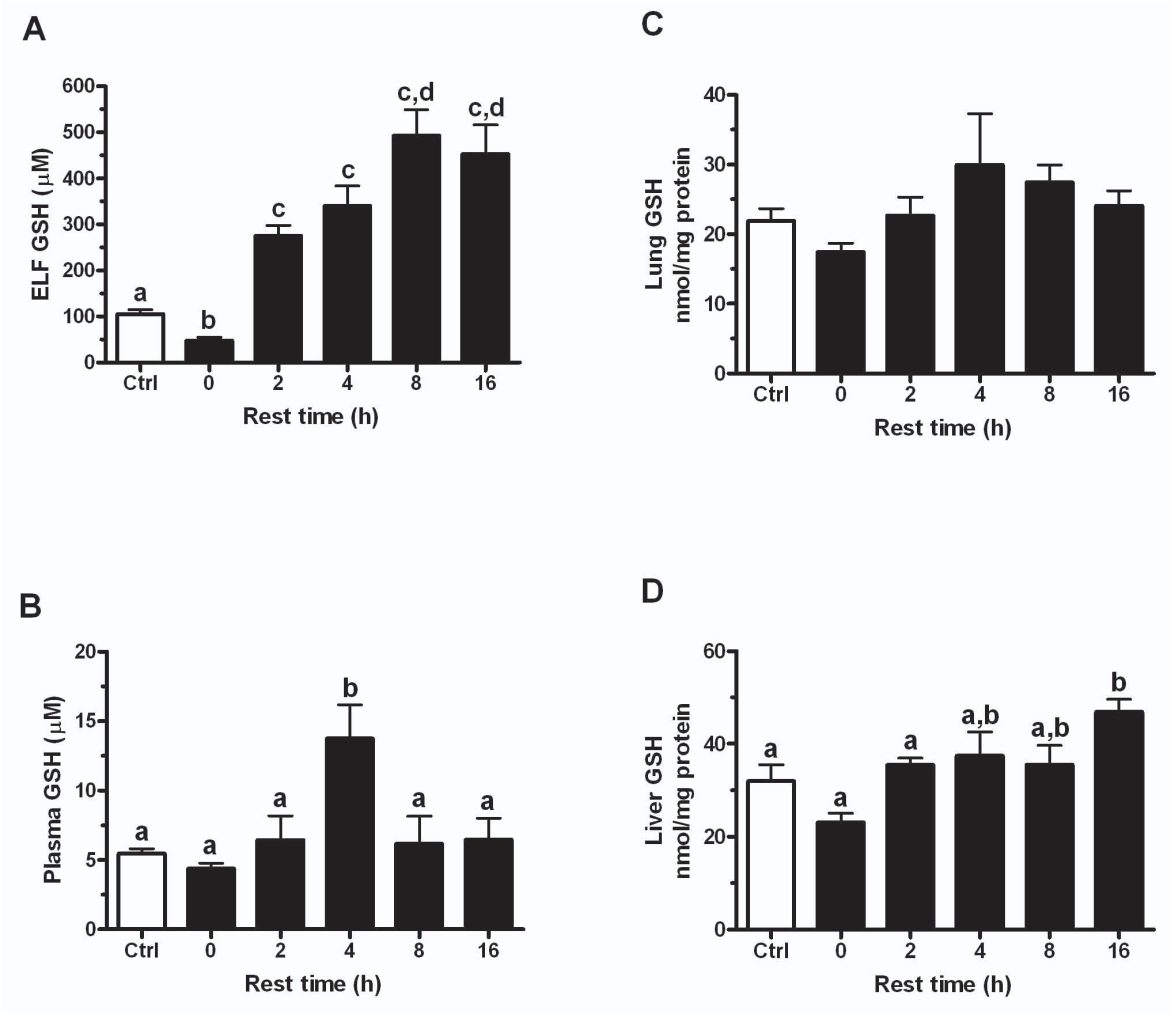
**GSH levels rebound during CS exposure recovery period**. The normal recovery period between smoke exposures is overnight which is typically about 16 hours. Mice were exposed to CS for 5 h and allowed to recover between 0 and 16 hours. ELF (A) GSH levels rebounds within 2 hours after exposure and peaks at 8 hours while plasma (C) GSH levels peak at 4 hours after CS exposure. The liver (D) GSH levels also rebound back to control levels and the lung (B) GSH levels are unchanged. Data represented as mean ± SEM, with different letters representing statistically significant differences between groups, p < 0.05.

**Figure 5:**
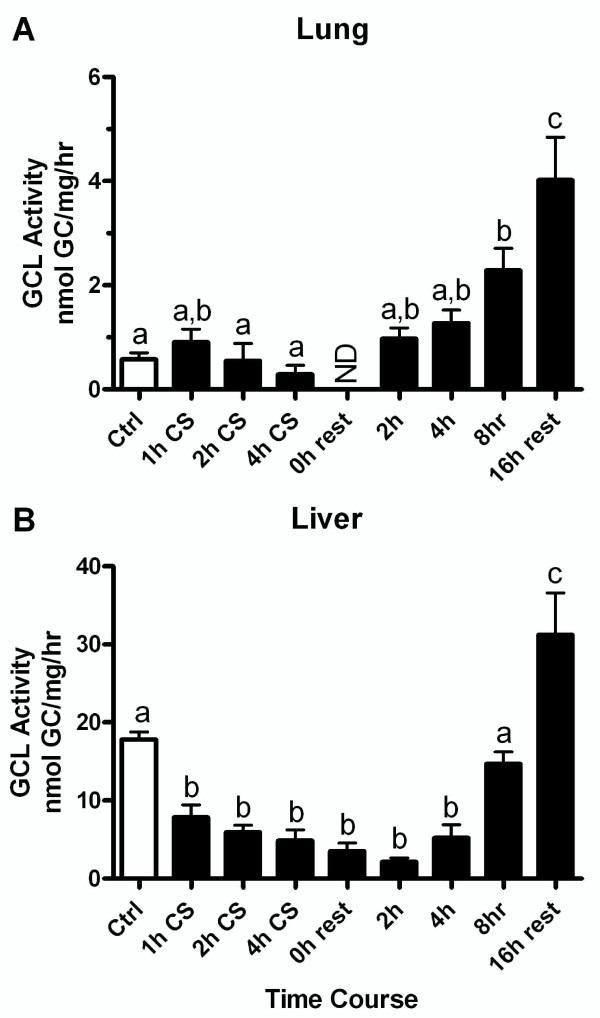
**GSH synthesis increases during the CS exposure recovery periods in both the lung and liver**. γ-Glutamylcysteine ligase (GCL) is the rating limiting step in GSH synthesis and it was measured in the lung (A) and liver (B) at multiple time points during the CS exposure and during the recovery period. Data represented as mean ± SEM, with different letters representing statistically significant differences between groups, p < 0.05.

Lung ELF GSH levels were reduced immediately following the CS exposure, yet after the first day of exposure to CS the GSH levels greatly rebound during the rest periods. The rest period between CS exposures was typically overnight, generally about 16 hours. To investigate this rebound period, mice were exposed to CS for 5 hours and then allowed to rest in a room receiving filtered air for 0, 2, 4, 8, or 16 hours. The GSH levels in the ELF (Figure 4A), plasma (Figure 4C), and liver (Figure 4D) were slightly reduced immediately following the CS exposure with no rest period, while the lung (Figure 4B) was not changed. As expected the GSH levels rebound during the rest period, with the ELF GSH levels rising to about 250 μM within the first 2 hours. Additionally, the depleted plasma and liver levels also rebound back to control levels. Intriguingly the plasma GSH levels spike to about 13 μM at 4 hours while the ELF GSH peaks at nearly 500 μM by 8 hours. These changes were mirrored by changes in the lung (Figure 5A) and liver (Figure 5B) tissue GCL activity which is a good indicator of a tissue's capacity to maintain GSH levels. This data shows that while the GSH was initially depleted by CS, the adaptive response was initiated and within 2 hours GSH levels return to normal and then rose above normal levels.

### GSH adaptive response prevents CS induced GSH depletion

**Figure 6:**
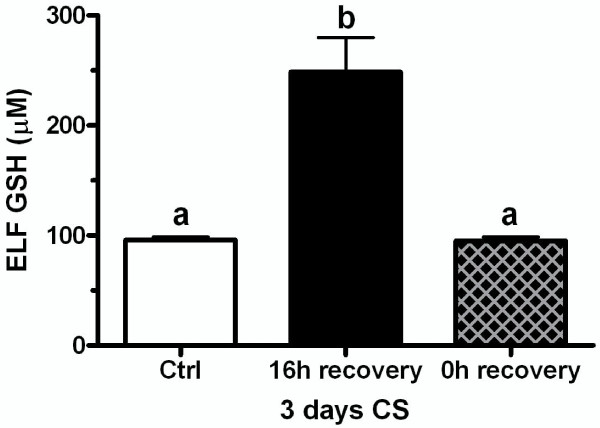
**The maintained GSH adaptive response in the ELF prevents CS induced GSH depletion below basal levels**. The maintained GSH adaptive response in the ELF occurs within 3 days. Mice were exposed to CS for 3 days to induce GSH adaptive responses and ELF GSH levels were analyzed after either 16 hours of recovery (closed bar) or immediately following the CS exposure (crossed bar). Data represented as mean ± SEM, with different letters representing statistically significant differences between groups, p < 0.05.

There appears to be two distinct phases of the GSH adaptive response to CS exposures starting with an initial GSH adaptive response and then a maintained GSH response in the ELF. The ELF GSH levels that occur with the initial adaptive response are roughly 500-600 μM (Figure 1A and 4A) while the GSH levels for the maintained adaptive response over repeated CS exposures are only about 250-300 μM (Figure 1A). We sought to determine whether the maintained adaptive level was sufficient to prevent CS induced GSH depletion upon repeated CS exposures. Mice were exposed to CS for 3 days to achieve an adaptive GSH level of roughly 250 μM and they were sacrificed immediately after the third day of CS exposure or after 16 hours of rest (Figure 6). The ELF GSH levels after the rest period was the expected 250 μM but when the mice were sacrificed immediately following the third day of CS exposure the ELF GSH levels were no different than control levels unlike the naïve mice which CS exposure depleted the ELF GSH levels by 50% (Figure 4A). This data suggests that the ELF GSH level of the maintained adaptive response is calibrated to prevent the CS-induced depletion of GSH levels below basal levels.

## Discussion

COPD is a disease that takes decades to develop. Not all smokers develop COPD, but of those that do, the vast majority are over 60 years old [19]. Both COPD and lung cancer show a separation in the time between the exposure and actual disease symptoms, which for COPD can be 30 years or more. This is one of the largest challenges to understanding the effects of CS and how it leads to COPD. Among researchers studying CS-induced lung damage the most common animal model of chronic exposure uses a smoking regime of 5 hours per day, 5 days per week. This regimen has been shown to be effective at causing airway enlargement in rodents after at least 6 full months of CS exposure [20,21]. However, little is known about the early changes that allow the lung to be able to withstand the oxidant insult of CS for such a long time. Many acute studies utilize a completely different exposure pattern with very high CS concentrations, which is not necessarily comparable to the smoking regimen used for chronic studies [22,23]. In the present study we have examined GSH changes using the most common rodent exposure regimen for chronic CS studies and have shown that there is a GSH adaptive response to CS that occurs very early on and can be maintained for periods of up to 4 months.

Many of the acute changes that occur with CS are routinely overlooked. Most of the focus is on the chronic effects of CS, with the rationale that COPD is a disease that arises from chronic smoking. It is well established that humans can smoke for years without any major health complications, while rodents can be exposed for months on end without any ill effect. Additionally, only modest effects on airspace enlargement occur in mice after 6 months of CS exposure [24]. While it is well known that the lung can tolerate CS for extended periods of time, very little is known about the mechanisms the lung uses for adaptation.

GSH is one of the most ubiquitous low molecular weight antioxidants in the body, it is expressed at high levels within cells and is found at relatively high levels in some extracellular fluids such as the lung ELF [4] and bile [25]. In the present study, two phases of the GSH adaptive response to CS exposure were observed, an initial exaggerated adaptive response and a maintained adaptive response. Similar to the responses that occur with other exposures, GSH is initially increased to very high levels in the ELF with CS exposure. This response may be part of an emergency response with the sudden onslaught of oxidants from the CS exposure or it may be the depletion of GSH during the CS exposure combined with the increase in oxidants from CS that acts as a trigger to initiate this response. Similar responses in GSH adaptive responses have been observed in the liver with acetaminophen poisoning [26].

One of the more interesting aspects is that while the CS exposure depletes the ELF, plasma, and liver GSH levels, the lung tissue GSH levels remain unchanged. This may be due to the lung utilizing GSH from the plasma, in turn causing the liver to export more GSH into the plasma. The ability of the lung to utilize GSH from the plasma seems evident based on the fact that during the rest period the plasma GSH spikes at 4 hours while at the same time point the lung GSH is slightly higher and the ELF GSH is still rising. The similarity in changes in GCL activity in the liver with the rebound increase in ELF GSH also point to the liver as a potential source for this GSH. Potentially the lung may be utilizing GSH from the plasma through an intact uptake mechanism shown to occur in the lung [27], which is then apically transported into the ELF, resulting in no net change in lung tissue GSH but a drastic increase in ELF GSH. These data suggests that GSH in the ELF may in fact originate from the plasma and hints at a strong reliance of the lung on the inter-organ supply of GSH especially during oxidative stress.

The GSH response with repeated exposures is also very interesting since the level of GSH in the ELF is established at a lower, but still protective level within the first 3 days of CS exposure. More importantly, comparable levels are maintained for at least 4 months that suggests these adaptive responses are established early and can be maintained for extended periods of time. This may also give insight into why rodents, and potentially humans, can tolerate CS for long periods of time. It is conceivable that all or part of the adaptive response is broken or defective early in the disease process or becomes insufficient due to age-related decline in GSH levels [16].

Another interesting facet of the maintained adaptive response is that the GSH level of roughly 250-300 μM may not be arbitrary. It seems as though the amount of GSH depletion that occurs during the CS exposures may trigger the amount of GSH that is maintained in the ELF between exposures. This suggests that there is some component in the ELF or lung that can act as a sensor to titrate the increase in GSH levels to prevent the ELF GSH level from dropping below basal levels. Some of the possible factors that are abundant in CS are polyaromatic hydrocarbons (PAHs) that are inducers of cytochrome P450s and are well known sources of reactive oxygen species [28]. This source of oxidative stress could maintain elevated levels of GSH observed with CS exposures. Another potential regulator of the chronic GSH adaptive response to CS exposure are the glutathione-S-transferases (GSTs) that conjugate GSH to xenobiotics for transport that could stimulate an increase in GSH synthesis to match GSH transport. Some GSTs are actually protective against cigarette smoke induced damage [29]. Carbon monoxide (CO) levels are high in CS and CO is a known inhibitor of heme-containing enzymes such as the cytochrome P450s [30] and mitochondrial complexes [31]. CO has well recognized protective effects in models associated with oxidative stress [32] and is a known inducer of GCL [33]. In terms of actual lung disease, a possible scenario may be that symptoms, including excessive inflammation and oxidative stress, may be tied to these adaptive responses and may only begin to arise when these adaptive responses fail.

There are many studies that have examined the link between antioxidants and inflammatory and oxidative pathways. ELF GSH levels have been shown to be inversely correlated with proinflammatory cytokine release both in vitro and in vivo [16,34]. Furthermore, the depletion of GSH has been widely shown to exacerbate oxidative stress and oxidative modifications of molecules [35-37]. GSH is a critically important molecule in both direct reactions with oxidants as well as several enzymatic detoxification pathways. Despite this, there are instances where the ability to synthesize GSH, and in turn establish an adaptive response is compromised. For instance, the normal aging process results in impaired GSH synthesis capability resulting in as much as a 50% reduction in available GSH in tissues and fluids [16]. Interestingly, aging is an independent risk factor for COPD [38]. The age-related decline in the ability to synthesize GSH results in not only lower basal levels of GSH, but also a decreased adaptive response to CS. Moreover, aged mice have been shown to be more susceptible to the release of proinflammatory cytokines and DNA oxidation from the same CS exposure than young mice with proper robust adaptive responses [16]. A number of genes involved in GSH synthesis, utilization, and transport have been shown to have polymorphisms associated with many lung diseases including COPD. For instance, a number of variants in the GCL gene have been associated with cystic fibrosis [39], and COPD [40]. The GSTs are enzymes responsible for conjugating xenobiotics with GSH and also are commonly associated with lung diseases including COPD [41]. In addition, the only known transporter that places GSH in the ELF is CFTR, the gene defective in cystic fibrosis [42]. These findings, along with the fact that the lung can normally maintain GSH for long periods even with the oxidant burden of CS, lend credence to the theory that disease symptoms could arise when adaptive responses fail or are dysfunctional.

## Conclusions

The present study has shown that many of the changes in GSH with regard to CS occur very acutely and can be maintained for longer periods of time. Furthermore, the ELF GSH levels that are maintained may in fact be tied to the CS induced depletion of GSH and that the lung may utilize GSH originating from other sources such as the liver and plasma to generate the high levels of ELF GSH. These data provide more insight into the acute effects of CS and how the lung resists the oxidant effects of CS. Examining the adaptive processes that are established to respond to the initial CS burden may help to understand what pathways become defective or are compromised and may contribute to the pathogenesis of COPD.

## Abbreviations

BAL: Bronchoalveolar lavage; BALF: Bronchoalveolar lavage fluid; COPD: chronic obstructive pulmonary disease; CS: cigarette smoke; CSE: cigarette smoke extract; ELF: epithelial lining fluid; GSH: glutathione;

## Competing interests

The authors declare that they have no competing interests

## Authors' contributions

NSG wrote the manuscript and performed the experiments and analyzed the samples. EM and SG helped with obtaining in vivo samples and processing. RJM provided oversight and help with cigarette smoke exposures and study design. BJD conceived of the study and helped write the manuscript. All authors have read and accepted the final manuscript
